# Palmitic acid induces intestinal lipid metabolism disorder, endoplasmic reticulum stress and inflammation by affecting phosphatidylethanolamine content in large yellow croaker *Larimichthys crocea*

**DOI:** 10.3389/fimmu.2022.984508

**Published:** 2022-08-19

**Authors:** Wei Fang, Yongtao Liu, Qiuchi Chen, Dan Xu, Qiangde Liu, Xiufei Cao, Tingting Hao, Lu Zhang, Kangsen Mai, Qinghui Ai

**Affiliations:** ^1^ Key Laboratory of Aquaculture Nutrition and Feed (Ministry of Agriculture and Rural Affairs) and Key Laboratory of Mariculture (Ministry of Education), Ocean University of China, Qingdao, China; ^2^ Tongwei Co., Ltd., Chengdu, China; ^3^ Healthy Aquaculture Key Laboratory of Sichuan Province, Chengdu, China; ^4^ Laboratory for Marine Fisheries Science and Food Production Processes, Qingdao National Laboratory for Marine Science and Technology, Qingdao, China

**Keywords:** intestinal homeostasis, palmitic acid, phosphatidylethanolamine, lipid metabolism, ER stress, inflammatory response

## Abstract

In the 21^st^ century, intestinal homeostatic imbalance has emerged as a growing health challenge worldwide. Accumulating evidence reveals that excessive intake of saturated fatty acid (SFA) induces intestinal homeostatic imbalance. However, the potential molecular mechanism is still unclear. In the present study, we found that palm oil or palmitic acid (PA) treatment disturbed lipid metabolism homeostasis and triggered endoplasmic reticulum (ER) stress and inflammation in the intestine or intestinal cells of large yellow croaker (*Larimichthys crocea*). Interestingly, PA treatment significantly decreased phosphatidylethanolamine (PE) content in the intestinal cells. PE supplementation decreased triglyceride content in the intestinal cells induced by PA treatment by inhibiting fatty acid uptake and lipogenesis. PE supplementation suppressed ER stress. Meanwhile, PE supplementation alleviated inflammatory response through p38 MAPK-p65 pathway, reducing the damage of intestinal cells caused by PA treatment to some extent. Our work revealed that intestinal homeostatic imbalance caused by PA treatment was partly due to the decrease of PE content. PE consumption might be a nutritional strategy to regulate intestinal homeostasis in fish and even human beings.

## Introduction

Effective function of intestine is important in regulating physiology, metabolism, and immunity in the whole body (1). Current studies have demonstrated that the intestinal homeostasis is closely related to multiple factors, including genetic mutations, environment, gut microbiota and dietary factors (2). With the rapid urbanization of developed and developing countries, excessive Western diet consumption which is rich in statured fatty acid (SFA) is one of the severe challenges to intestinal homeostasis. Previous study has demonstrated that intake of palmitic acid (PA) could induce inflammatory cytokine production (3) and lipid metabolism disorder (4) in the intestinal cells. However, the underlying mechanism is not well understood.

Phospholipid is not only an essential component of intestine, but also an important signal molecule, which is involved in maintaining metabolic and immune homeostasis (5). Increasing studies have demonstrated that phospholipid treatment inhibited proinflammatory gene expression *via* direct inhibition of NF-κB in intestinal epithelial cells (6, 7). Moreover, clinical study has revealed that phospholipid supplementation could alleviate inflammatory response in those patients with ulcerative colitis (8). Ethanolamine which is the base constituent of phosphatidylethanolamine (PE) is required for the intestinal development and promotes intestinal functions (9). Our previous study has found that PA treatment induced adverse effects that might be associated with PE content in macrophage (10), which is one of the most abundant phospholipid in cells (11). Thus, we hypothesized that PE metabolism may be involved in PA-induced intestinal homeostatic imbalance.

Fish are the largest group of vertebrates in the world (12). Although fish are less evolved than mammals, the nutrient-sensing and immunity are conservated to some extent (13). In aquaculture, different nutritional components can influence fish health by affecting the intestinal homeostasis (14–16), while the potential molecular mechanisms are still poorly understood. The method of isolating and culturing intestinal cells of fish are mature (17). Therefore, fish are good model animals to study the pathogenesis of intestinal homeostatic imbalance (12, 18). Accordingly, the present study investigated the mechanism of PA on lipid metabolism and immune homeostasis in the intestine and aimed to develop nutritional strategies to promote intestinal homeostasis of fish and human beings.

## Materials and methods

### Animal ethics

All animal experiments in the present were carried out in strict standard operation with the Management Rule of Laboratory Animals (Chinese Order No. 676 of the State Council, revised March 1, 2017).

### Diet formulation, fish culture and sample collection

Two diets were formulated in the present study as follows: a fish oil diet (FO) and a palm oil diet (PO) (**Table 1**) (19). Healthy juvenile large yellow croaker (average weight 15.8 ± 0.14 g) was obtained from Ningde, China. After two weeks of domestication, total fish were randomly divided into two groups (three seawater cages in each group). Fish were fed twice a day for 10 weeks. At end of the feeding trial, fish were anaesthetized with MS-222 (Sigma, USA), then intestines of fish were collected for further study.

**Table 1:** Formulation and proximate composition of experimental diets (% dry weight) (19).

Diets		
Ingredients^a^	Fish oil (FO)	Palm oil (PO)
White fish meal^b^	35	35
Soybean meal^b^	28	28
Wheat meal^b^	23.8	23.8
Soybean lecithin	1.5	1.5
Vitamin premix^c^	2	2
Mineral premix^c^	2	2
Attractant mixture^d^	0.1	0.1
Mold inhibitor^e^	0.1	0.1
Fish oil	7.5	0
Palm oil	0	7.5
Total	100.00	100.00
Proximate analysis (dry matter %)		
Crude protein	42.23	42.37
Crude lipid	12.05	12.53

a These ingredients were supplied by Great Seven Biotechnology Co., Ltd, China.

b Fish meal (dry mater, %): 70.55% crude protein and 7.21% crude lipid, Soybean meal (dry mater, %): 51.89% crude protein and 1.16% crude lipid, Wheat meal (dry mater, %): 15.09% crude protein and 0.15% crude lipid.

c The mixture of mineral mixture and vitamin mixture according to Yan et al. (20).

d Attractant: the mixture of glycine acid (50%) and betaine (50%).

e Mold inhibitor: the mixture of calcium propionic acid (50%) and fumaric acid (50%).

### Cell culture and treatment

Intestinal cells were isolated and cultured in the six-well plates in DMEM/F12 medium with 15% fetal bovine serum (BI, Israel) at 27°C and 5% CO_2_ atmosphere, according to our previous study (17). To investigate the effects of PA on lipid metabolism, ER stress and inflammation in the intestinal cells, cells were treated with 100 μM PA (Sigma, USA) for 24h. To prove the function of PE in the intestinal cells, cells were co-treated with 10uM PE (Sigma) and 100uM PA for 24h.

### Detection of triglyceride and PE content

The content of TG in the intestine and intestinal cells were measured by a commercial kit (Applygen, China), according to our previous study (21). Meanwhile, the content of PE was measured using an ELISA kit (Fankew, China), according to manufacturer’s instructions.

### Gene expression quantification

Intestine and cell samples were processed for total RNA extraction using TRIzol reagent (Takara, Japan). Genomic DNA was removed at 42 °C for 2 min using the PrimeScript™ RT reagent kit (Takara), and then cDNA was synthesized. The program of cDNA synthesis consisted of 37°C for 15 min and 85°C for 5 s. Relative gene expression was performed with quantitative real-time polymerase chain reaction (qRT-PCR) using SYBR kit (Takara) and calculated by the method according to a previous study (22). β-actin was used as the internal reference gene. The primers for qRT-PCR were listed in the **Table 2**.

**Table 2:** Primer sequences used for quantitative real-time PCR in the study.

Genes	Forward	Reverse
*cd36*	CAGGCAGTTCTGGTTATTTGATTTG	GCAGCAGGAAGGAGACAGTGTTATT
*fatp1*	CAACCAGCAGGACCCATTACG	CATCCATCACCAGCACATCACC
*fatp4*	TCAACGACCGAGGTGGAGGG	CGGAAGGAAGCGGAGGAACA
*fabp2*	GGGTCACCTTTGAGTACAGCCTTG	CCTTCTTGAAAATCCTCTTTGCGT
*fabp3*	CCAAACCCACCACTATCATCTCAG	GCACCATCTTTCCCTCCTCTATTG
*srebp1c*	TCTCCTTGCAGTCTGAGCCAAC	TCAGCCCTTGGATATGAGCCT
*scd1*	AAAGGACGCAAGCTGGAACT	CTGGGACGAAGTACGACACC
*acc1*	GACTTGGCGGAATACCTACTGG	GCTTGCTGGATGATCTTTGCTT
*acc2*	AAAGAATCCCTGTGCAGGCTGTC	TCCTCCTCGGTCCAATCCACTC
*dgat1*	GGTATCTTGGTGGACCCCATTCA	TGAGCACCGTGGCTGAAGGAAAGA
*dgat2*	TTCGGTGCTTTCTGCAACTTCG	AAGGATGGGGAAGCGGAAGT
*adrp*	CAAGGCTAATGCGTTGGAAGA	AGTTGAGCGGCGTGTTATTGA
*pparα*	GTCAAGCAGATCCACGAAGCC	TGGTCTTTCCAGTGAGTATGAGCC
*cpt1α*	GCTGAGCCTGGTGAAGATGTTC	TCCATTTGGTTGAATTGTTTACTGTCC
*aco*	AGTGCCCAGATGATCTTGAAGC	CTGCCAGAGGTAACCATTTCCT
*mtp*	CTTGAGTCGCTGATTGCTGC	TGAGGTCGCTGTAACCCTTG
*apob48*	AGAGTGTTGTCCAGGATAAAGATGC	CAGGGCTCAGGGTCTCAGTC
*sar1b*	GCATGACTTTCACCACCTTTG	GTTCTGCTTTTGATTCTCCCA
*sec13*	CTCCTTCTATTGGTCTCCCC	ACAGCGTCACCTTGTTGTCT
*sec31*	CTGGTGGAGAAGGTGGTGGT	GTGTTGTCGGGCAGGTAGGT
*sec23*	ACACCAGTCATACCTACCGC	AGATCCTCAAACTCTTCCCC
*sec24*	TCCCCAGCGACAGATTTCTA	TTGGTGCAGCGTATCCTCAT
*il-1β*	CATAGGGATGGGGACAACGA	AGGGGACGGACACAAGGGTA
*il-6*	CGACACACCCACTATTTACAAC	TCCCATTTTCTGAACTGCCTCT
*il-8*	AATCTTCGTCGCCTCCATTGT	GAGGGATGATCTCCACCTTCG
*cox2*	CTGGAAAGGCAACACAAGC	CGGTGAGAGTCAGGGACAT
*grp78*	GGTGGCGATGACAAGCAAAC	CTGAGAACAGCAGCAACAAGC
*xbp1*	GTCTTCTGAGTCCGCAGCAGGTG	AGGATGTCCAGAATGCCCAGTAG
*atf4*	GCCGTTATTCTGCTCCATCTTCT	AGACCTTACCCTGAGCCCACAT
*atf6*	CAGATAATAAGGAGGCTGAGAGTGC	CGTAGGTATGATGAGGTGCGTAGT
*chop*	TCTGGATGTTCTGGAGAGTTGTTC	AGGATGATGATGAGGTGTGATGC
*pisd*	TCCTTTCCAACTCGCCTCCTCTC	GTAAATCCTCCACCGCTGCTTCC
*selenoi*	CTGTTCCTTTACCGTGACCTTACCC	CAGGCTGCTGTGCTTCAGAGTG
*etnk1*	CAATGAGTTTGCGGGCTTGAATGAG	CCTCCACCTCTCCGTCAGTCAC
*etnk2*	TTCACCGACCAAGCATCCAACATC	AGAGCACCACAGGAGAGCCAAG
*pcyt2*	GCAAGACGGAGGTGATTCCAGAC	CACTGTCGATGGTCCTGAAGATTCC
*β-actin*	GACCTGACAGACTACCTCATG	AGTTGAAGGTGGTCTCGTGGA

### Western blotting analysis

Intestine and cell samples were homogenized on ice using RIPA lysis buffer (Solarbio, China). Then the homogenate was centrifuged at 4°C for 10 min to obtain supernatant. The protein supernatant was separated on 10% SDS-PAGE and transferred into PVDF membranes. Membranes were blocked with 5% skimmed milk and incubated with specific primary antibodies at 4°C overnight. The primary antibodies were listed in the **Table S1**. After that, the membranes were incubated with secondary antibody (Golden Bridge, China) and visualized by using an electrochemiluminescence kit (Beyotime, China).

### Data analysis

All data in the present study were performed with SPSS 17.0 software (IBM, USA) by using independent sample t-test or one-way analysis of variance (ANOVA) followed by Tukey’s test. All data were expressed as mean ± standard deviation (SD). The level of significance was set at P < 0.05.

## Results

### Effect of PO or PA treatment on lipid metabolism in the intestine or intestinal cells

**Figure 1:**
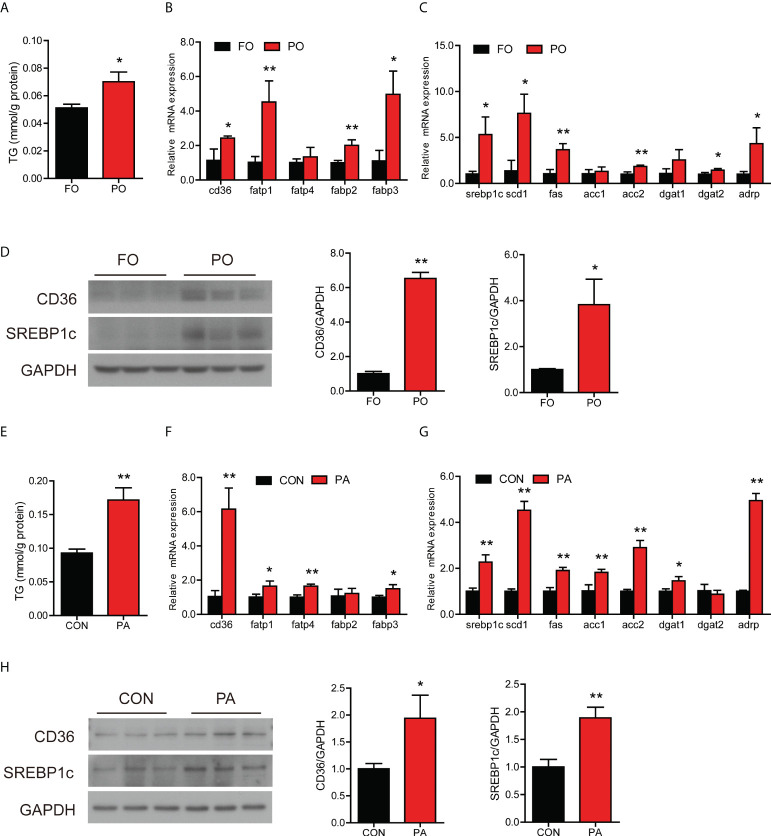
Effect of PO or PA treatment on lipid metabolism in the intestine or intestinal cells of large yellow croaker. **(A)** TG content in the intestine after different diets (n = 3). **(B)** Fatty acid uptake and **(C)** lipid synthesis-related genes expression in the intestine after different diets (n = 3). **(D)** Protein levels of CD36 and cleavage of SREBP1c in the intestine after different diets (n = 3). **(E)** TG content in the intestinal cells after BSA or PA treatment (n = 3). **(F)** Fatty acid uptake and **(G)** lipid synthesis-related genes expression in the intestinal cells after BSA or PA treatment (n = 3). **(H)** Protein levels of CD36 and cleavage of SREBP1c in the intestinal cells after BSA or PA treatment (n = 3). Results were presented as mean ± standard deviation (SD) and analyzed using independent *t*-test (*P < 0.05, **P < 0.01). FO, fish oil; PO, palm oil; CON, bovine serum albumin treatment; PA, palmitic acid treatment.

Compared to the control group, the content of TG in the intestine was significantly higher in the PO group (**Figure 1A**). The mRNA expression of fatty acid uptake-related genes in the intestine was significantly increased in the PO group, including *cd36*, *fatp1*, *fabp2* and f*abp3* (**Figure 1B**). Dietary PO significantly up-regulated the mRNA expression of lipid synthesis-related genes (**Figure 1C**). Meanwhile, the mRNA expression of chylomicron assembly and secretion-related genes, including *apob48*, *sar1b*, and *sec23* was basically up-regulated in the PO group (**Figure S1A**). Dietary PO significantly increased the mRNA level of *aco* which plays a vital role in fatty acid β-oxidation (**Figure S1B**). Moreover, the protein levels of CD36, cleavage of SREBP1c, SAR1B, CPT1α and PPARα in the PO group were significantly higher than those in the control group (**Figure 1D** and **Figure S1C**), while the protein levels of APOB48 and SEC13 were not remarkably changed (**Figure S1C**). Thus, dietary PO induced intestinal abnormal lipid accumulation in large yellow croaker through disturbing intestinal lipid homeostasis.

To further investigate whether PO disturbed intestinal lipid metabolism, we treated the intestinal cells with PA *in vitro* experiment. PA treatment significantly induced TG accumulation in cells (**Figure 1E**). The mRNA expression of *cd36*, *fatp1*, *fatp4* and *fabp3* was significantly increased in the PA group than the control group (**Figure 1F**), which suggested that PA treatment promoted fatty acid uptake in cells. Meanwhile, the transcription of genes related to lipid synthesis was significantly increased after PA treatment (**Figure 1G**). In terms of lipid secretion, the gene expression showed an upward trend after PA treatment (**Figure S1D**). In addition, PA treatment significantly upregulated the gene expression involved in fatty acid β-oxidation, including *pparα*, *aco* and *cpt1α* (**Figure S1E**). Moreover, the protein levels of CD36, cleavage of SREBP1c, APOB48, CPT1α and PPARα were significantly increased after PA treatment (**Figure 1H**, **Figure S1F**). These results above indicated that PO or PA treatment induced abnormal lipid accumulation in the intestine or intestinal cells of large yellow croaker through disturbing lipid homeostasis.

### Effect of PO or PA treatment on ER stress in the intestine or intestinal cells

**Figure 2:**
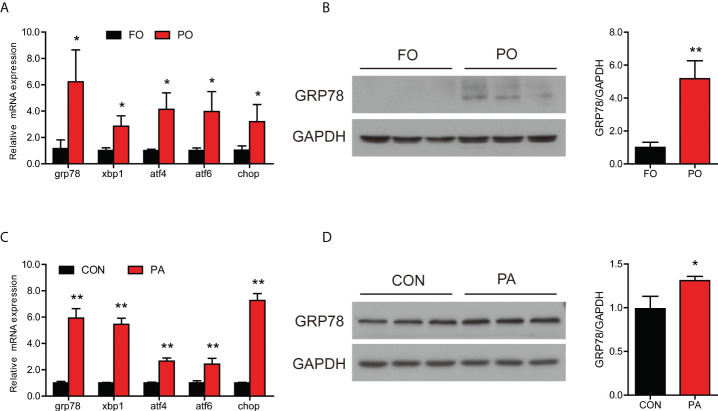
Effect of PO or PA treatment on ER stress and UPR pathway in the intestine or intestinal cells of large yellow croaker. **(A)** Relative mRNA expression of ER stress-related genes in the intestine after different diets (n = 3). **(B)** Protein level of GRP78 in the intestine after different diets (n = 3). **(C)** Relative mRNA expression of ER stress-related genes in the intestinal cells after BSA or PA treatment (n = 3). **(D)** Protein level of GRP78 in the intestinal cells after BSA or PA treatment (n = 3). Results were presented as mean ± standard deviation (SD) and analyzed using independent *t*-test (*P < 0.05, **P < 0.01). FO, fish oil; PO, palm oil; CON, bovine serum albumin treatment; PA, palmitic acid treatment.

The transcription of *grp78*, *xbp1*, *atf4*, *atf6* and *chop* was higher in the PO group (**Figure 2A**). Moreover, PO replacement significantly upregulated the protein level of GRP78 compared to the control group (**Figure 2B**).

Consistent with experiments *in vivo*, incubation of intestinal cells with PA significantly increased the transcription of *grp78*, *xbp1*, *atf4*, *atf6* and *chop* (**Figure 2C**). Moreover, the protein level of GRP78 was significantly increased after PA treatment (**Figure 2D**). These results suggested that PO or PA treatment induced ER stress in the intestine or intestinal cells of large yellow croaker.

### Effect of PO or PA treatment on inflammation in the intestine or intestinal cells

**Figure 3:**
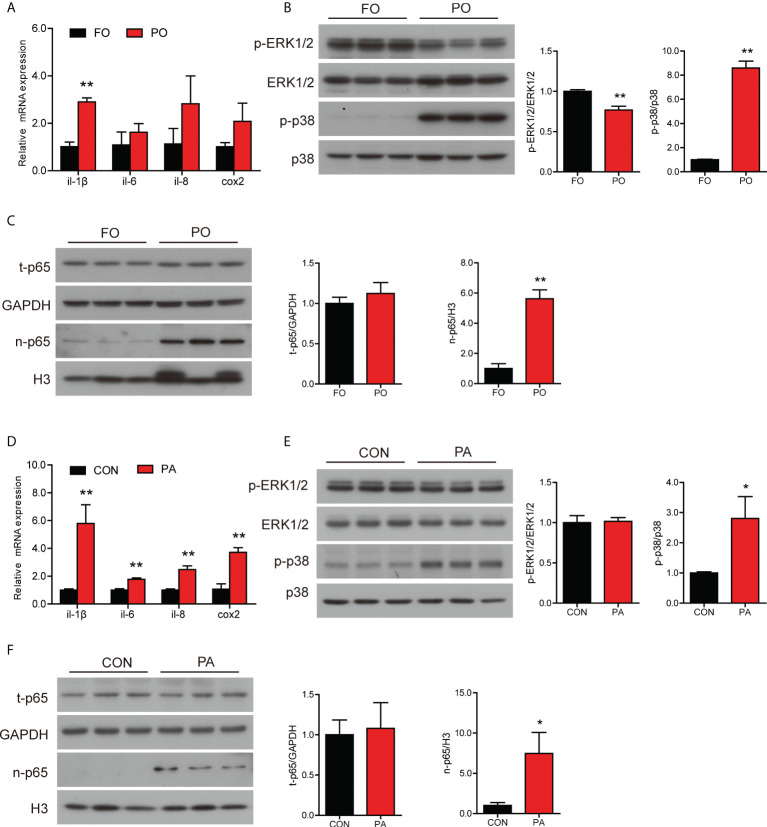
Effect of PO or PA treatment on inflammation in the intestine or intestinal cells of large yellow croaker. **(A)** Relative mRNA expression of proinflammatory genes in the intestine after different diets (n = 3). **(B)** Protein levels of p-ERK1/2, ERK1/2, p-p38 MAPK and p38 MAPK in the intestine after different diets (n = 3). **(C)** Protein levels of total p65 and nuclear p65 in the intestine after different diets. **(D)** Relative mRNA expression of proinflammatory genes in the intestinal cells after BSA or PA treatment (n = 3). **(E)** Protein levels of p-ERK1/2, ERK1/2, p-p38 MAPK and p38 MAPK in the intestinal cells after BSA or PA treatment (n = 3). **(F)** Protein levels of total p65 and nuclear p65 in the intestinal cells after BSA or PA treatment (n = 3). Results were presented as mean ± standard deviation (SD) and analyzed using independent *t*-test (*P < 0.05, **P < 0.01). FO, fish oil; PO, palm oil; CON, bovine serum albumin treatment; PA, palmitic acid treatment.

The mRNA level of *il-1β* was significantly higher and the gene expression of *il-6*, *il-8* and *cox2* followed an upward trend in the PO group compared to the FO group (**Figure 3A**). We further examined the protein levels involved in inflammation and MAPK pathway. Dietary PO significantly upregulated the ratio of p38 MAPK to MAPK and decreased the ratio of p-ERK1/2 to ERK1/2 (**Figure 3B**). Meanwhile, the protein level of nuclear p65 was significantly increased in the PO group (**Figure 3C**).

Next, we further measured the gene and protein levels related to inflammation in the intestinal cells with PA treatment. The transcription of proinflammation genes was significantly higher in the PA group, including *il-1β*, *il-6*, *il-8* and *cox2* (**Figure 3D**). Results of western blotting analysis were similar to those *in vivo*. The ratio of p38 MAPK to MAPK was significantly higher after PA treatment, while the ratio of p-ERK1/2 to ERK1/2 was not remarkably changed (**Figure 3E**). Moreover, the protein level of nuclear p65 was significantly rose in the PA group (**Figure 3F**). Thus, these results showed that PO or PA treatment induced inflammation in the intestine or intestinal cells of large yellow croaker.

### Effect of PO or PA treatment on the content of PE in the intestine or intestinal cells

**Figure 4:**
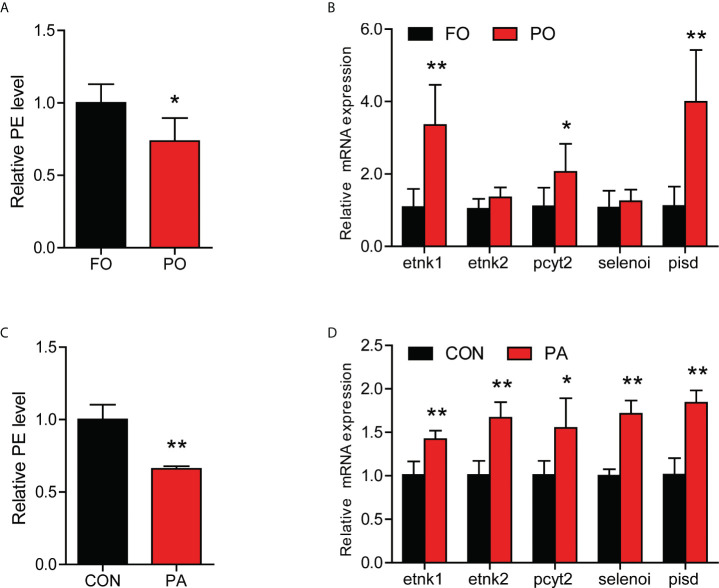
Effect of PO or PA treatment on the content of PE in the intestine or intestinal cells of large yellow croaker. **(A)** PE content in the intestine after different diets (n = 3). **(B)** Relative mRNA expression of PE synthesis related-genes in the intestine after different diets (n = 3). **(C)** PE content in the intestinal cells after BSA or PA treatment (n = 3). **(D)** Relative mRNA expression of PE synthesis related-genes in the intestinal cells after BSA or PA treatment (n = 3). Results were presented as mean ± standard deviation (SD) and analyzed using independent *t*-test (*P < 0.05, **P < 0.01). FO, fish oil; PO, palm oil; CON, bovine serum albumin treatment; PA, palmitic acid treatment.

Our previous had found that PA-induced inflammation in macrophage might be related to the decrease of PE content (10). Next, we measured the content of PE in the intestinal cells *in vivo* and *in vitro*. The content of PE was significantly decreased in the PO group (**Figure 4A**), while the expression of genes related to PE synthesis were significantly upregulated, including *etnk1*, *pcyt2* and *pisd* (**Figure 4B**). Consistence with *in vivo* results, PA treatment significantly reduced the level of PE in the intestinal cells (**Figure 4C**), while the gene expression of *etnk1*, *etnk2*, *pcyt2*, *selenoi* and *pisd* were significantly upregulated (**Figure 4D**). These results above indicated that PO or PA treatment decreased the content of PE in the intestine or intestinal cells of large yellow croaker.

### Addition of PE alleviated the damage of intestinal cells caused by PA treatment to some extent

**Figure 5:**
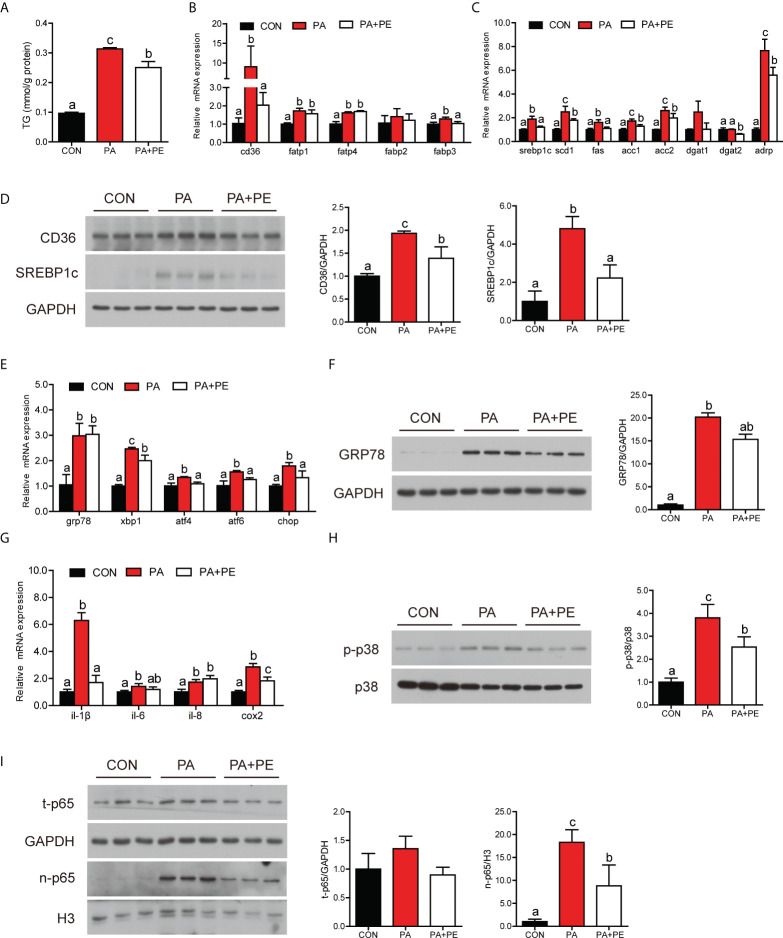
The addition of PE alleviated the damage of intestine cells caused by PA treatment to some extent. **(A)** TG content in the intestinal cells after different treatments (n = 3). **(B)** Fatty acid uptake and **(C)** lipid synthesis-related genes expression in the intestinal cells after different treatments (n = 3). **(D)** Protein levels of CD36 and cleavage of SREBP1c in the intestinal cells after different treatments (n = 3). **(E)** Relative mRNA expression of ER stress-related genes in the intestinal cells after different treatments (n = 3). **(F)** Protein level of GRP78 in the intestinal cells after different treatments (n = 3). **(G)** Relative mRNA expression of proinflammatory genes in the intestinal cells after different treatments (n = 3). **(H)** Protein levels of p-p38 MAPK and p38 MAPK in the intestinal cells after different treatments (n = 3). **(I)** Protein levels of total p65 and nuclear p65 in the intestinal cells after different treatments (n = 3). Results were presented as mean ± standard deviation (SD) and analyzed using one-way analysis of variance (ANOVA) followed by Tukey’s test (values without the same letter indicate significant difference among three treatments *P* < 0.05). CON, bovine serum albumin treatment; PA, palmitic acid treatment; PA+PE, palmitic acid and phosphatidylethanolamine co-treatment.

We speculated that PA treatment might disorder lipid metabolism and immune homeostasis by affecting the content of PE in the intestinal cells. To confirm the hypothesis intestinal cells were co-treatment with PA and PE. Compared to PA group, addition of PE significantly decreased the content of TG in the intestinal cells (**Figure 5A**). The transcription of fatty acid uptake-related genes was down-regulated in the PA+PE group, including *cd36* and f*abp3* (**Figure 5B**). The mRNA expression of lipid synthesis-related genes was down-regulated in the PA+PE group, including *srebp1c*, *scd1*, *fas*, *acc1*, *acc2*, *dgat2* and *adrp* (**Figure 5C**). We also found that PA+PE treatment significantly decreased the gene expression of lipid secretion and β-oxidation (**Figures S2A, B**). Moreover, the protein levels of CD36, cleavage of SREBP1c, APOB48 were significantly decreased in the PA+PE group compared to PA group (**Figure 5D** and **Figure S2C**). However, the protein levels of SAR1B, SEC13, CPT1α and PPARα were not remarkably changed (**Figure S2C**). In addition, we also found that PE supplementation significantly decreased the mRNA levels of genes related to ER stress, including *xbp1*, *atf4*, *atf6* and *chop* (**Figure 5E**). Moreover, compared to the PA group, the protein level of GRP78 was significantly decreased in the PA+PE group (**Figure 5F**). In term to inflammation, addition of PE could down-regulate proinflammation gene expression including *il-1β* and *cox2* (**Figure 5G**). Compared to the PA group, the protein levels of p-P38 MAPK and nuclear p65 were significantly decreased in the PA+PE group (**Figures 5H, I**). Thus, these results indicated that addition of PE could alleviate the damage of intestinal cells induced by PA treatment to some extent.

## Discussion

Excessive dietary SFA consumption is linked to many metabolic diseases, including nonalcoholic fatty liver disease (23), atherosclerosis (24), and type 2 diabetes (25). However, the mechanism of SFA in the intestinal lipid metabolism and immune homeostasis is not clear. In the present study, we found that dietary PO induced intestinal abnormal lipid accumulation in large yellow croaker through disturbed the intestinal lipid metabolism homeostasis. The lipid metabolism in the intestine of fish is similar to that of mammals, including dietary fatty acid uptake, *de novo* lipogenesis, CM secretion and fatty acid β-oxidation (26). The gene expression of fatty acids uptake was increased in the PO group, which indicated that dietary PO induced massive fatty acids and monoacylglycerol (MAG) to be absorbed into the intestine. Fatty acids were transported to ER and reconverted to TG. As expected, the gene and protein levels of lipid synthesis was significantly increased in the PO group. Thus, we speculated that dietary fatty acid uptake and *de novo* lipogenesis may be the main factors which induced intestinal abnormal lipid accumulation. These results were consistent with previous study that overfeeding palm oil promoted visceral and hepatic fat storage (27). However, dietary PO also improved chylomicron secretion and β-oxidation in the intestine, which may be the self-regulation of the intestine to decrease excessive lipid accumulation. The data was consistent with previous study that β-oxidation was higher in the chicken fed with a PO diet than those Fed with a FO diet (28). Consistent with the results *in vivo*, PA treatment disturbed lipid metabolism in the intestinal cells. Overall, these results showed that dietary PO disturbed the balance of intestinal lipid metabolism, resulting in excessive intestinal lipid deposition in large yellow croaker.

The ER is a critical site of lipid metabolism (29). Our previous study has demonstrated that the impairment of ER function disordered intestinal lipid metabolism in large yellow croaker (17). Thus, we speculated that dietary PO might affect ER homeostasis in the intestine. In this study, the data demonstrated that PO or PA treatment induced ER stress *in vivo* or *in vitro*, which were consistent with previous studies in mammals (30–32). Accumulating studies have suggested that excessive SFA accumulation in the ER could affect calcium homeostasis of ER leading to destroy folding capacity of ER (33, 34). Moreover, SFA increases production of metabolic intermediates like ceramides (35) and reactive oxygen species (36) which induce ER stress *via* disruption of ER structure and function resulting in ER stress. The mechanism of PO or PA treatment induced ER stress in the intestine or intestinal cells needs to be further studied.

Previous studies have shown that excessive lipid accumulation and ER stress are usually accompanied by inflammatory response (37, 38). Excessive production of proinflammatory cytokines is the main cause of intestinal injury. In the present study, we found that dietary PO enhanced the nuclear p65 level and proinflammation gene expression, which was consistent with previous study in mammals (39). Next, we measured the expression of the MAPK pathway, which is known as a crucial mediator of inflammation (40). The ratio of p-p38 MAPK to p38 MAPK was increased in the PO group, while the phosphorylation level of ERK1/2 was decreased. The obtained results *in vitro* were almost consistent with those *in vivo*. Previous studies have demonstrated that p38 MAPK was necessary to regulate p65 and induced proinflammation gene expression (41, 42). Thus, we speculated that PO might induce intestinal inflammation *via* p38 MAPK-p65 pathway in large yellow croaker.

Our previous study has found that PA treatment significantly decreased the content of PE in the macrophage cells (10). PE is the second most abundant phospholipid in mammal cells, which is not only simple component of the membrane (43), but also essential for many cellular processes such as protein folding (44), autophagy (45), and oxidative phosphorylation (46). Thus, we hypothesized that PA disordered the lipid metabolism and immune homeostasis may be involved in the content of PE in the intestinal cells. In the present study, we found that PO or PA treatment significantly decreased the content of PE *in vivo* or *in vitro*. However, the PE synthesis related genes expression was increased, which may be negative feedback for the decrease of PE content. Previous study has demonstrated that elimination of the CDP-ethanolamine pathway which is the main pathway to form PE triggers fatty acid synthesis, leading to liver steatosis (47). As we expected, incubation with PE could alleviate the TG abnormal accumulation induced by PA treatment to some extent through decreasing fatty acid uptake and lipogenesis. We also found that addition of PE down-regulated gene expression related to UPR pathway, leading to relieve ER stress. The result was consistence with the previous study that inhibiting the PERK-CHOP signaling pathway to protect cells from ER stress-induced damage (48). Moreover, PE supplementation could inhibit inflammation induced by PA treatment. Similarly, dietary eicosapentaenoic acid in the form of PE chronic inflammation *via* the inhibition of NF-κB activation in obese adipose tissue (49). Concomitant with the above previous report, the present study indicated that addition of PE decreased the levels of proinflammation genes *via* inhibiting the p38 MAPK-p65 pathway. Moreover, a recent study has found that PE metabolism affected T_FH_ cell differentiation and humoral immunity (50). Thus, PE intake might be a nutritional strategy to regulate intestinal homeostasis in fish and even human beings.

**Figure 6:**
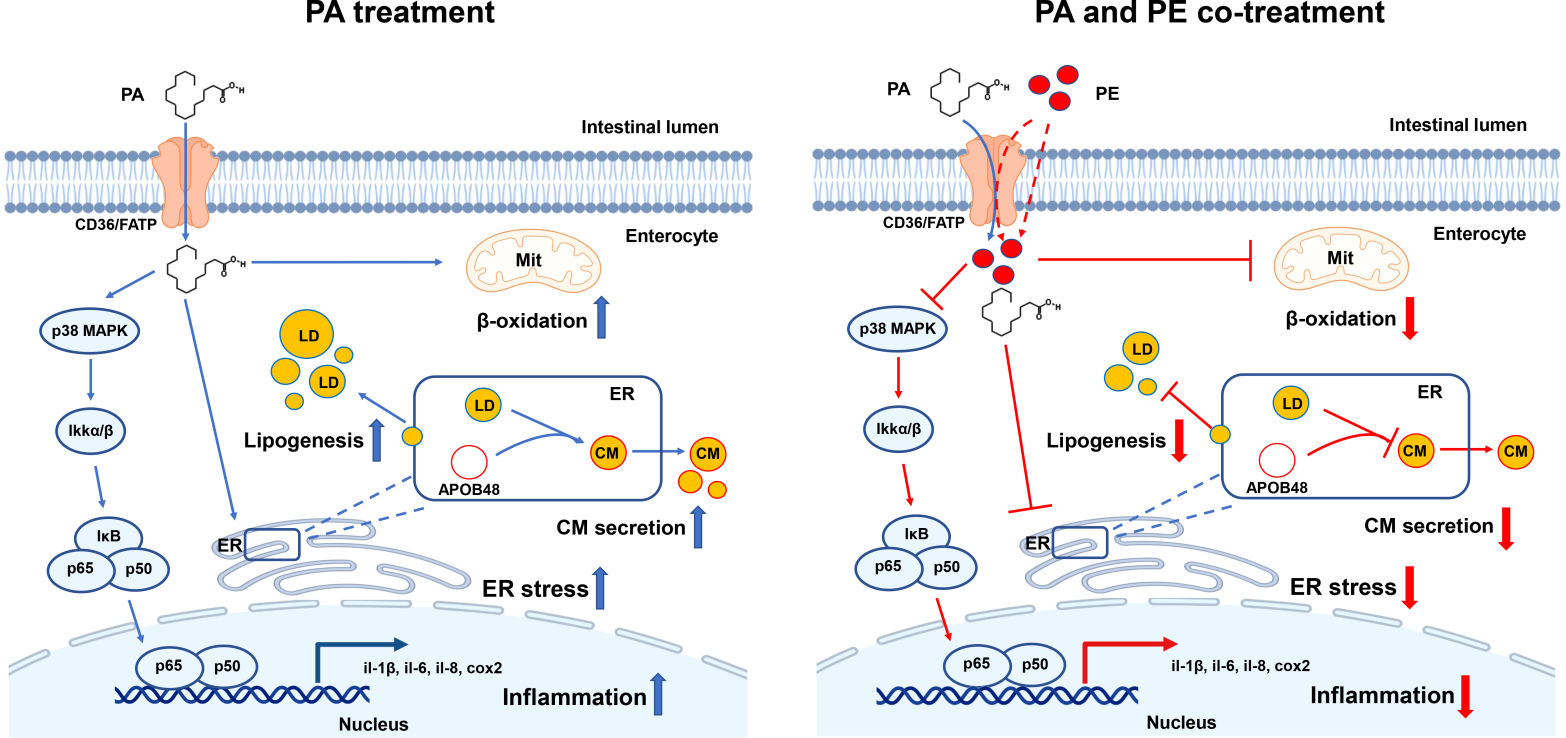
A working model showed that PA treatment disrupted lipid metabolism homeostasis and triggered ER stress and inflammation in the intestinal cells of large yellow croaker, while addition of PE could alleviate the damage of intestinal cells induced by PA treatment to some extent.

In conclusion, for the first time, the present study demonstrated that PA treatment induced lipid metabolism disorder, ER stress and inflammation in the intestine, which was associated with decreasing the content of PE. Addition of PE could alleviate the damage of intestinal cells caused by PA treatment to some extent (**Figure 6**). PE consumption might be a nutritional strategy to reduce the use of drugs in aquaculture regulating intestinal homeostasis in fish, which contributes to the production of green and safe food. In addition, the key genes of PE metabolism might be targets to maintain intestinal health of human beings.

## Data availability statement

The original contributions presented in the study are included in the article/**Supplementary Material**. Further inquiries can be directed to the corresponding author.

## Ethics statement

The animal study was reviewed and approved by the Management Rule of Laboratory Animals (Chinese Order No. 676 of the State Council, revised March 1, 2017).

## Author contributions

WF, and QA designed the experiments. WF performed the main experiments and wrote the original draft. YL, QC, and XC conducted other experiments. DX, and QL. analyzed the data. LZ, TH, and KM revised the manuscript. All authors contributed to the final editing and approval of the manuscript.

## Funding

This research is supported by the Key Program of National Natural Science Foundation of China (Grant no.31830103), the earmarked fund for CARS-47, and the Scientific and Technological Innovation of Blue Granary (grant no. 2018YFD0900402).

## Acknowledgments

We thank Jikang Shentu, Lin Huang and Shengwei Xu for providing experimental animals and facilities. We also appreciate Jianlong Du, Jiamin Li, Xiaojun Xiang, Xueshan Li for providing antibodies.

## Conflict of interest

Author LZ was employed by company Tongwei Co., Ltd.

The remaining authors declare that the research was conducted in the absence of any commercial or financial relationships that could be construed as a potential conflict of interest.

## Publisher’s note

All claims expressed in this article are solely those of the authors and do not necessarily represent those of their affiliated organizations, or those of the publisher, the editors and the reviewers. Any product that may be evaluated in this article, or claim that may be made by its manufacturer, is not guaranteed or endorsed by the publisher.

## Glossary

**Table:**

SFA	saturated fatty acid
PA	palmitic acid
ER	endoplasmic reticulum
PE	phosphatidylethanolamine
FO	fish oil diet
PO	palm oil diet
TG	triglyceride
TBST	tris bufflered saline with Tween
cd36	fatty acid translocase
fatp1	fatty acid transport protein 1
fatp4	fatty acid transport protein 4
fabp1	fatty acid-binding protein 1
fabp2	fatty acid-binding protein 2
fabp3	fatty acid-binding protein 3
srebp1c	sterol regulatory element binding protein 1 c
scd1	stearoyl-CoA desaturase 1
acc1	acetyl-CoA carboxylase 1
acc2	acetyl-CoA carboxylase 2
dgat1	diacylglycerol acyltransferase 1
dgat2	diacylglycerol acyltransferase 2
adrp	adipose differentiation-related protein
ppara	peroxisome proliferator-activated receptor alpha
cpt1a	carnitine palmitoyl transferase 1 alpha
aco	acyl-CoA oxidase
mtp	microsomal triglyceride transfer protein
apob	apolipoprotein
sar1b	secretion associated Ras related GTPase 1B
sec13	sec13 homolog nuclear pore and COPII coat complex component
sec31	sec31 homolog A COPII coat complex component
sec23	sec23 homolog A coat complex II component
sec24	sec24 homolog A COPII coat complex component
il-1b	interleukin-1 beta
il-6	interleukin-6
il-8	interleukin-8
cox2	cyclooxygenase 2
grp78	glucose related protein 78
xbp1	X-box binding protein 1
atf4	activating transcription factor 4
atf6	activating transcription factor 6
chop	C/EBP homologous protein
pisd	phosphatidylserine decarboxylase
selenoi	selenoprotein I
etnk1	ethanolamine kinase 1
etnk2	ethanolamine kinase 2
pcyt2	phosphate cytidylyltransferase 2
b-actin	beta-actin
