# Supplementary material for: Palmitic acid induces intestinal lipid metabolism disorder, endoplasmic reticulum stress and inflammation by affecting phosphatidylethanolamine content in large yellow croaker Larimichthys crocea
Source: Front Immunol. 2022 Aug 19;13:984508. doi: 10.3389/fimmu.2022.984508 (PMC9437641; doi:10.3389/fimmu.2022.984508)
Supplement: Supplementary file 1 [file DataSheet_1.docx]

**Table S1** Antibodies used for Western blot in the study

| Antibodies | Source | Identifier |
| --- | --- | --- |
| Rabbit monoclonal Anti-GRP78 | Cell Signaling Technology | CAT#3177 |
| Rabbit monoclonal Anti-ERK1/2 | Cell Signaling Technology | CAT#4695 |
| Rabbit monoclonal Anti-ERK1/2  (phospho Thr202/Tyr204) | Cell Signaling Technology | CAT#4370 |
| Rabbit monoclonal Anti-p38 MAPK | Cell Signaling Technology | CAT#8690 |
| Rabbit monoclonal Anti-p38 MAPK  (phospho Thr180/Tyr182) | Cell Signaling Technology | CAT#9215 |
| Rabbit monoclonal Anti-p65 | Cell Signaling Technology | CAT#8242 |
| Rabbit monoclonal Anti-SAR1B | Abcam | CAT#ab155278 |
| Rabbit polyclonal Anti-H3 | Abcam | CAT#ab1791 |
| Rabbit polyclonal Anti-PPARα | Absin | CAT#abs117362 |
| Mouse monoclonal Anti-SEC13 | Santa Cruz | CAT# sc-514308 |
| Rabbit polyclonal Anti-CPT1α | Proteintech | CAT#15184-1-AP |
| Rabbit polyclonal Anti-SREBP1 | Wan Lei Bio | CAT#WL02093 |
| Mouse polyclonal Anti-GAPDH | Golden Bridge Biotechnology | CAT#TA-08 |
| Rabbit polyclonal Anti-CD36 | This paper | N/A |
| Rabbit polyclonal Anti-APOB48 | This paper | N/A |

**
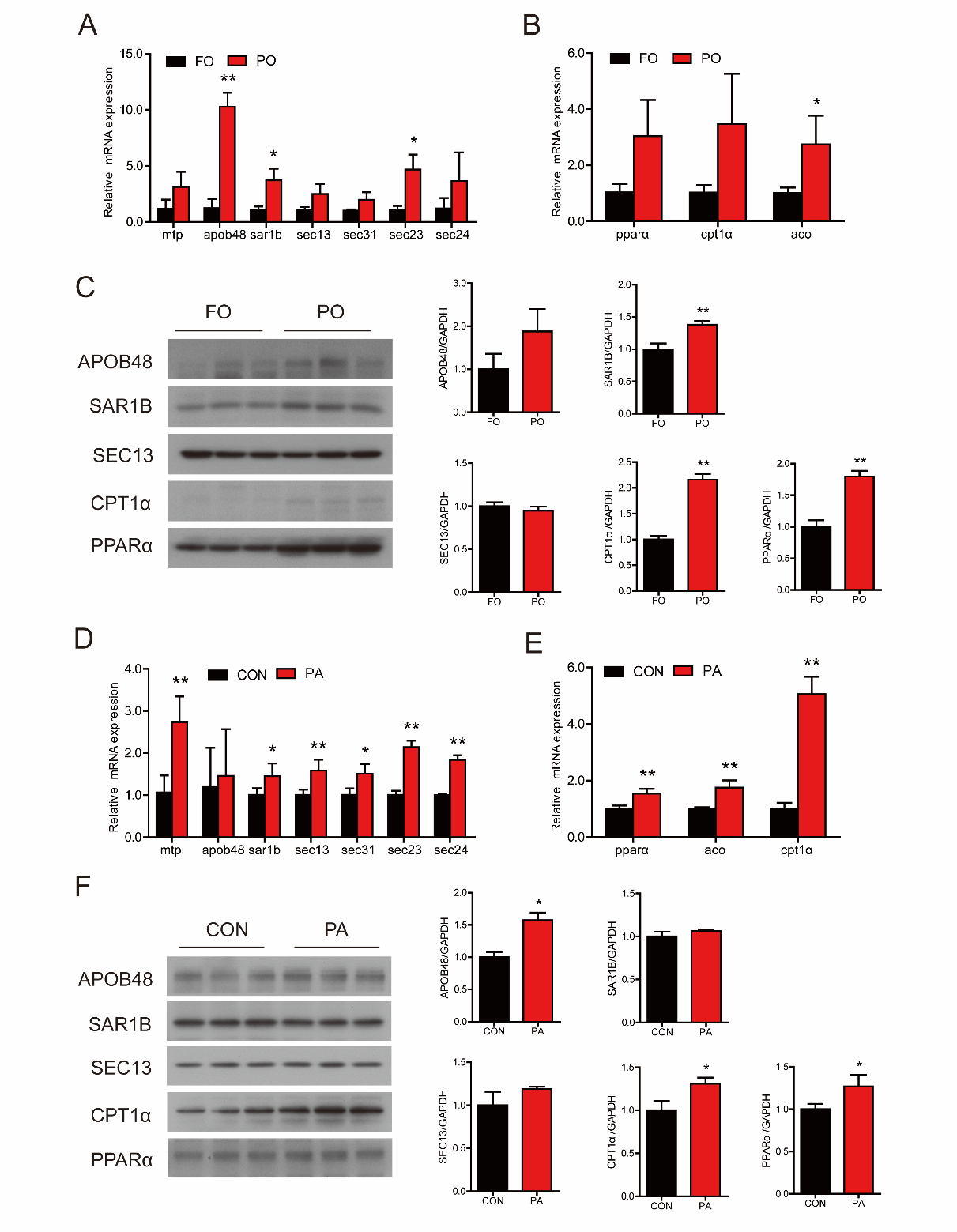
**

**Figure S1** Effect of PO or PA treatment on lipid metabolism in the intestine or intestinal cells of large yellow croaker, related to Figure 1. (A) Chylomicron secretion and (B) fatty acid β-oxidation related genes expression in the intestine after different diets (n=3). (C) Protein levels of APOB48, SAR1B, SEC13, CPT1α and PPARα in the intestine after different diets (n=3). (D) Chylomicron secretion and (E) fatty acid β-oxidation related genes expression in the intestinal cells after BSA or PA treatment (n=3). (F) Protein levels of APOB48, SAR1B, SEC13, CPT1α and PPARα in the intestinal cells after BSA or PA treatment (n=3). Results were presented as mean ± standard deviation (SD) and analyzed using independent *t*-test (**P* < 0.05, ***P* < 0.01). FO: fish oil, PO: palm oil, CON: bovine serum albumin treatment, PA: palmitic acid treatment.


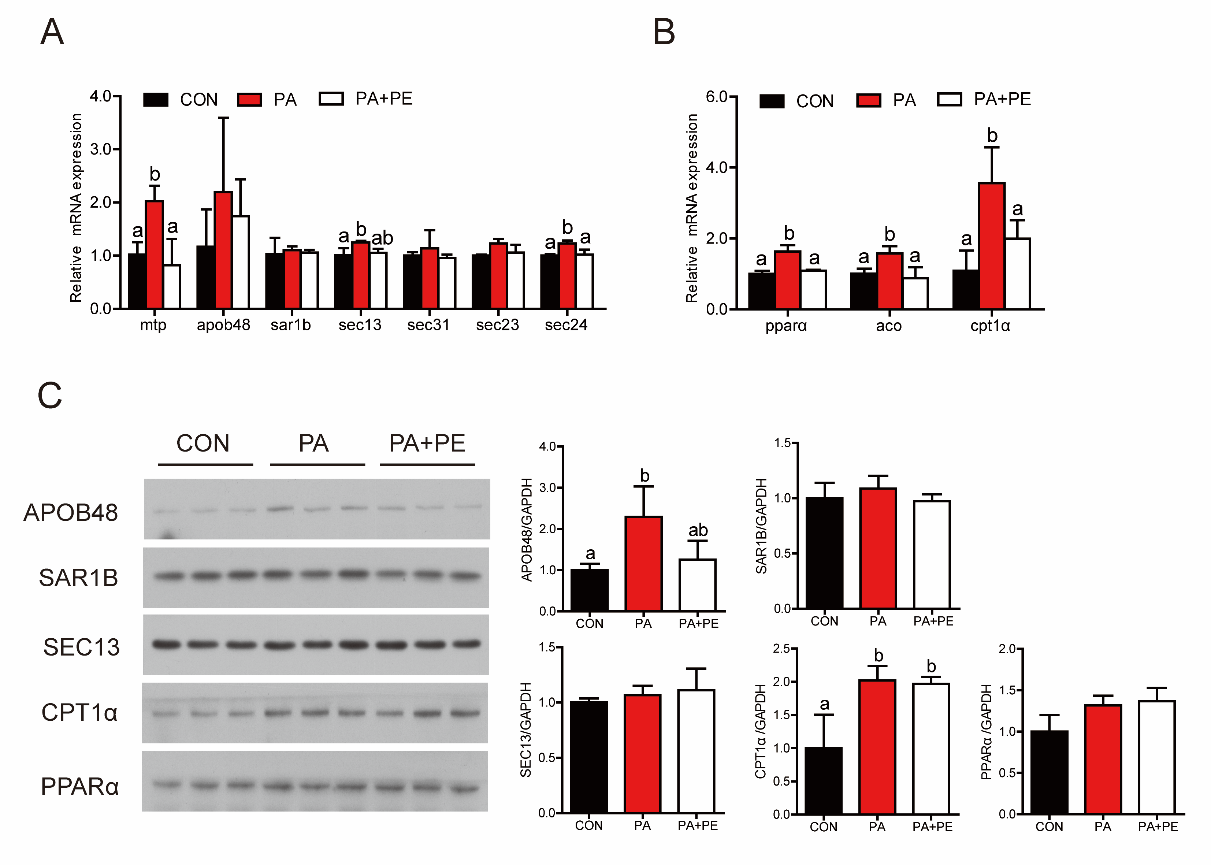


**Figure S2** The addition of PE alleviated the damage of intestine cells caused by PA treatment to some extent, related to Figure 5. (A) Chylomicron secretion and (B) fatty acid β-oxidation related genes expression in the intestinal cells after different treatments (n=3). (C) Protein levels of APOB48, SAR1B, SEC13, CPT1α and PPARα in the intestinal cells after different treatments (n=3). Results were presented as mean ± standard deviation (SD) and analyzed using one-way analysis of variance (ANOVA) followed by Tukey’s test (values without the same letter indicate significant difference among three treatments *P* < 0.05). CON: bovine serum albumin treatment, PA: palmitic acid treatment, PA+PE: palmitic acid and phosphatidylethanolamine co-treatment.
